# Procedural Characteristics and Clinical Outcomes Associated with Chronic Total Occlusion Percutaneous Coronary Intervention in Patients with a History of Prior Coronary Artery Bypass Graft: A Meta-Analysis

**DOI:** 10.31083/j.rcm2403089

**Published:** 2023-03-13

**Authors:** Songyuan He, Yingkai Li, Zichao Cheng, Yuchen Shi, Jinghua Liu

**Affiliations:** ^1^Center for Coronary Artery Disease (CCAD), Beijing Anzhen Hospital, Capital Medical University, Beijing Institute of Heart, Lung and Blood Vessel Diseases, 100029 Beijing, China

**Keywords:** percutaneous coronary intervention, chronic total occlusion, clinical outcomes

## Abstract

**Background:**

Owing to advances in procedural techniques and the training of interventional staff in catheterization labs, recent work has demonstrated the safety of percutaneous coronary intervention (PCI) as a treatment for patients suffering from chronic total occlusion (CTO). However, there has been little research focused on systematic comparisons of PCI outcomes in CTO patients that did or did not exhibit a history of previous coronary artery bypass grafting (CABG).

**Methods:**

Electronic databases were systematically searched for all studies comparing CTO-PCI outcomes for patients with and without a history of CABG, with event rates subsequently being compared via random-effects models with forest plots and odds ratios with 95% confidence intervals (CI), owing to the assumption of between-studies heterogeneity.

**Results:**

In total, 8 observational studies enrolling 13,509 CTO patients were identified, including 3389 and 10,120 patients with and without a history of prior CABG, respectively. Patients were enrolled in these studies from 1999–2018. Pooled analyses indicated that CABG history was not linked to a lower proportion of radial access 24 (95% CI 0.52–1.03, *p* = 0.08), and a prior CABG history was linked to a greater contrast volume (95% CI 0.12–0.44, *p *< 0.001), higher radiation dose (95% CI 0.27–0.40, *p *< 0.001), longer fluoroscopy time (95% CI 0.42–0.61, *p *< 0.001), longer procedural time (95% CI 0.38–0.64, *p *< 0.001), a higher number of implanted stents (95% CI 0.41–0.60, *p *< 0.001), longer total stent length (95% CI 0.21–0.60, *p *< 0.001), higher technical failure rates (95% CI 1.46–1.85, *p *< 0.001), and higher rates of procedural failure (95% CI 1.42–1.79, *p *< 0.001). The in-hospital mortality (95% CI 1.50–4.03, *p *< 0.001) and periprocedural mortality (95% CI 1.63–3.73, *p *< 0.001) of patients with a history of CABG were also higher. While stroke incidence was comparable in both groups (95% CI 0.80–4.47, *p* = 0.15), periprocedural major adverse cardiovascular and cerebrovascular events (MACCE) rates were significantly higher among patients exhibiting a history of CABG (95% CI 1.66–2.94, *p *< 0.001).

**Conclusions:**

These results suggest that CTO-PCI procedures may be more challenging and associated with lower rates of success in CABG patients relative to procedures performed in patients without any history of CABG. Moreover, in-hospital outcomes including MACCE and mortality were worse for patients that had undergone prior CABG.

## 1. Introduction

Advances in procedural techniques and instrumentation have contributed to substantial improvements in chronic total occlusion (CTO) percutaneous coronary intervention (PCI) safety and efficacy rates [1]. When successful, CTO-PCI procedures can offer an effective alternative treatment option for patients with a history of coronary artery bypass grafting (CABG) experiencing angina following bypass graft failure. However, prior pathological analyses have suggested that CTO lesions in CABG patients exhibit pronounced negative remodeling and calcification not evident in patients without such a history [2, 3]. Accordingly, some studies have suggested CABG to be a predictor of CTO-PCI procedural failure [4]. Moreover, the technical and procedural success rates for CTO-PCI have been reported to be lower in CABG patients without any corresponding increase in rates of in-hospital major complications [5, 6, 7]. However, further advances in the devices and materials used for these procedures have been developed since the publication of these prior studies, and it thus remains to be established as to whether these are associated with further improvements in clinical outcomes for treated patients. While one meta-analysis has explored this topic [8], it only analyzed four observational studies and there have since been several further published cohort studies, highlighting the need for an updated survey of the literature. This meta-analysis was thus performed with the goal of assessing procedural characteristics and clinical outcomes associated with CTO-PCI treatment of patients with and without a history of CABG.

## 2. Methods

### 2.1 Study Design

The Preferred Reporting Items for Systematic Reviews and Meta-Analyses (PRISMA) statement was used to direct this meta-analysis. The protocol of this meta-analysis had been registered on PROSPERO registration. The registration number is CRD42022373092.

### 2.2 Data Source and Search Strategy

The PubMed, Embase, and Cochrane Central databases were systematically and independently searched by two investigators (YCS and SYH) for all relevant studies comparing CTO-PCI procedural characteristics and clinical outcomes between patients that did and did not exhibit a history of CABG published as of August 15, 2021. Search terms included the following: (1) chronic total occlusion, CTO, and coronary occlusion; (2) percutaneous coronary intervention and PCI; (3) coronary artery bypass, coronary bypass, bypass surgery, and CABG. No age or language restrictions were placed on these studies. Relevant clinical trials were further identified by searching http://www.clinicaltrials.gov, as well as the proceedings of major international cardiology meetings (American College of Cardiology, European Society of Cardiology, American Heart Association, Transcatheter Cardiovascular Therapeutics, and The Society of Cardiovascular Angiography and Interventions). References of selected studies were additionally reviewed in an effort to identify relevant articles.

### 2.3 Study Selection

Studies exhibiting original CTO-PCI procedure-related for patients with or without a history of CABG that included cardiovascular event incidence as a primary outcome were included in this meta-analysis. No randomized data control trials (RCTs) relevant to this topic were identified. Case reports, case series, editorials, reviews, and abstracts without a corresponding full-text article were excluded from these analyses.

### 2.4 Data Extraction

Article titles and abstracts using the defined search strategy were independently reviewed by two investigators (YCS and SYH), with those articles meeting the inclusion criteria undergoing full-text review. Discrepancies were resolved by discussion and consensus. Evaluated procedural characteristics for included studies included radial access, contrast dosage, radiation dose and fluoroscopy time, procedural time, number of stents implanted and total stent length, technical failure, and procedural failure. Clinical complications included in this study were in-hospital death, periprocedural death, stroke, and periprocedural major adverse cardiovascular and cerebrovascular events (MACCE). Results were subject to sensitivity analyses to ensure the findings were robust, and study quality was assessed with the Newcastle-Ottawa scale for cohort studies.

### 2.5 Endpoints

The procedural characteristics of the enrolled studies included radial access, contrast dosage, radiation dose and fluoroscopy time, procedural duration, number and length of implanted stents, technical and procedural failure rates. Technical success was defined as successful CTO revascularization with achievement of <30% residual diameter stenosis within the treated segment and restoration of Thrombolysis in Myocardial Infarction grade 3 antegrade flow. Procedural success was defined as the achievement of technical success without any in-hospital complications. The clinical outcomes included in-hospital mortality, periprocedural mortality, stroke, and periprocedural MACCE incidence, which consisted of death, myocardial infarction (MI), and revascularization/stroke.

### 2.6 Data Analysis

The RevMan software program (version 5.4.1, The Cochrane Collaboration, London, United Kingdom) was used to conduct the present meta-analysis. Data were compared using random-effects models for all endpoints, with continuous variables being reported as pooled standardized mean difference (SMD) values and categorical values being reported with Mantel–Haenszel Odds Ratios (OR). All data pooled analysis results were reported with 95% confidence intervals (CI). Heterogeneity among studies was examined with Cochran’s Q test and *$I^{2}$*, corresponding to the percentage of overall variance attributable to heterogeneity rather than chance. Significant heterogeneity was considered present based on an *$I^{2}$*>50% and *p <* 0.05. Sensitivity analysis was performed to explore the effect of single research to the heterogeneity in this outcome. We visually examined funnel plot symmetry to assess publication bias.

## 3. Results

### 3.1 Study Characteristics

**Fig. 1.**
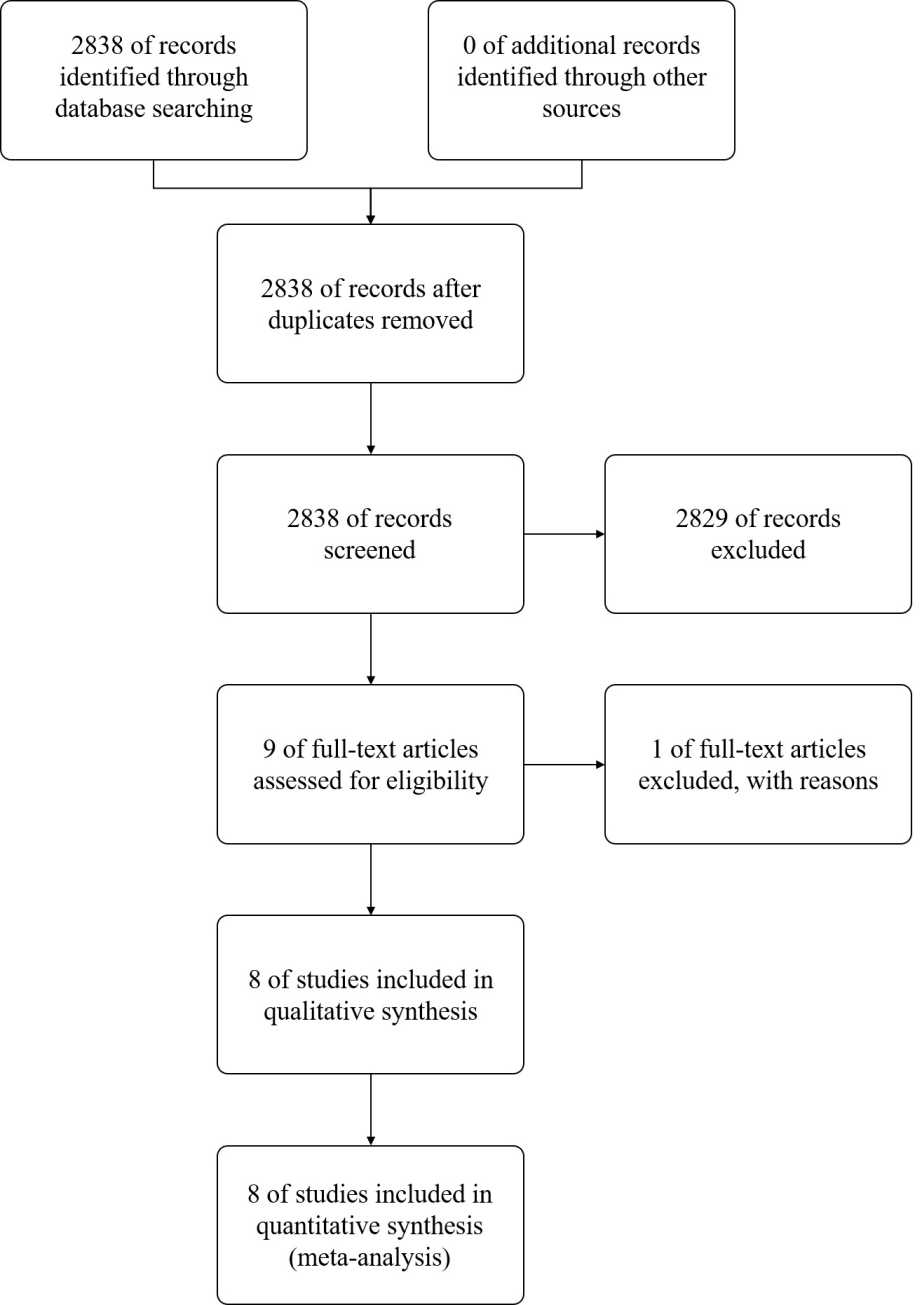
**Study selection flow chart**.

The PRISMA flow diagram corresponding to the study selection process for this meta-analysis is shown in Fig. 1. An initial search identified 2838 potentially relevant abstracts, of which 9 were subject to full-text review and 8 were ultimately enrolled in this study. These studies incorporated 13,439 patients, including 3349 and 10,090 with and without a history of CABG, respectively [5, 7, 9, 10, 11, 12, 13, 14]. All 8 studies were observational in design and had been published from 2013–2020, with follow-up durations ranging from 100 days to 32 months [5, 7, 9, 10, 11, 12, 13, 14]. Of these studies, 4 only assessed in-hospital outcomes [5, 7, 10, 13]. Details regarding study and patient characteristics are compiled in Table 1 [5, 7, 9, 10, 11, 12, 13, 14].

**Table 1.** **Study Characteristics**.

Study	Characteristics	Study total size (n)	Age (y)	Male sex (%)	Body mass index (kg/$m^{2}$)	Diabetes (%)	Dyslipidemia (%)	Hypertension (%)	Current smoker (%)	Previous myocardial infarction (%)	Previous PCI (%)
[Azzalini 2018] [9]	Prior CABG	401	69.2 ± 8.0	92	28.8 ± 5.1	48	91	87	12	56	73
	No prior CABG	1657	64.3 ± 10.6	87	28.6 ± 7.3	35	78	74	31	43	58
[Budassi 2021] [10]	Prior CABG	217	68.5 ± 8.5	86.2	28.3 ± 3.9	31.3	78.3	72.4	7.4	51.2	62.5
	No prior CABG	1035	64.9 ± 10.7	85.5	28.5 ± 4.8	25.5	64.7	59.3	24.6	36.6	55.9
[Dautov 2016] [11]	Prior CABG	175	70 ± 7	86	29 ± 5	52		93	7	65	76
	No prior CABG	295	64 ± 11	77	30 ± 6	30		75	23	51	67
[Michael 2013] [5]	Prior CABG	508	67.7 ± 9.0	86.2		44.3	96	92.6		44.9	43.4
	No prior CABG	855	63.3 ± 10.4	84.4		36.8	92.6	87.2		39.8	40.8
[Nikolakopoulos 2020] [12]	Prior CABG	502									
	No prior CABG	1082									
[Tajti 2019] [13]	Prior CABG	1101	67.3 ± 9.3	87.1	30.6 ± 5.8	48.80	95.30	93.70	20.50	56.40	73.60
	No prior CABG	2317	63.2 ± 10.2	83.8	30.7 ± 6.3	38.60	87.70	88.00	29.80	42.80	60.10
[Teramoto 2014] [7]	Prior CABG	153	68.2 (62.4–74.6)	82		42	35	59	18		
	No prior CABG	1139	66.0 (58.2–73.6)	82		37	37	61	25		
[Toma 2016] [14]	Prior CABG	292	68 ± 9	88	28.5 ± 4.4	39	91	90	7	48	23
	No prior CABG	1710	65 ± 11	83	28.1 ± 4.4	28	85	81	22	21	14

### 3.2 Procedural Characteristics

#### 3.2.1 Radial Access

**Fig. 2.**
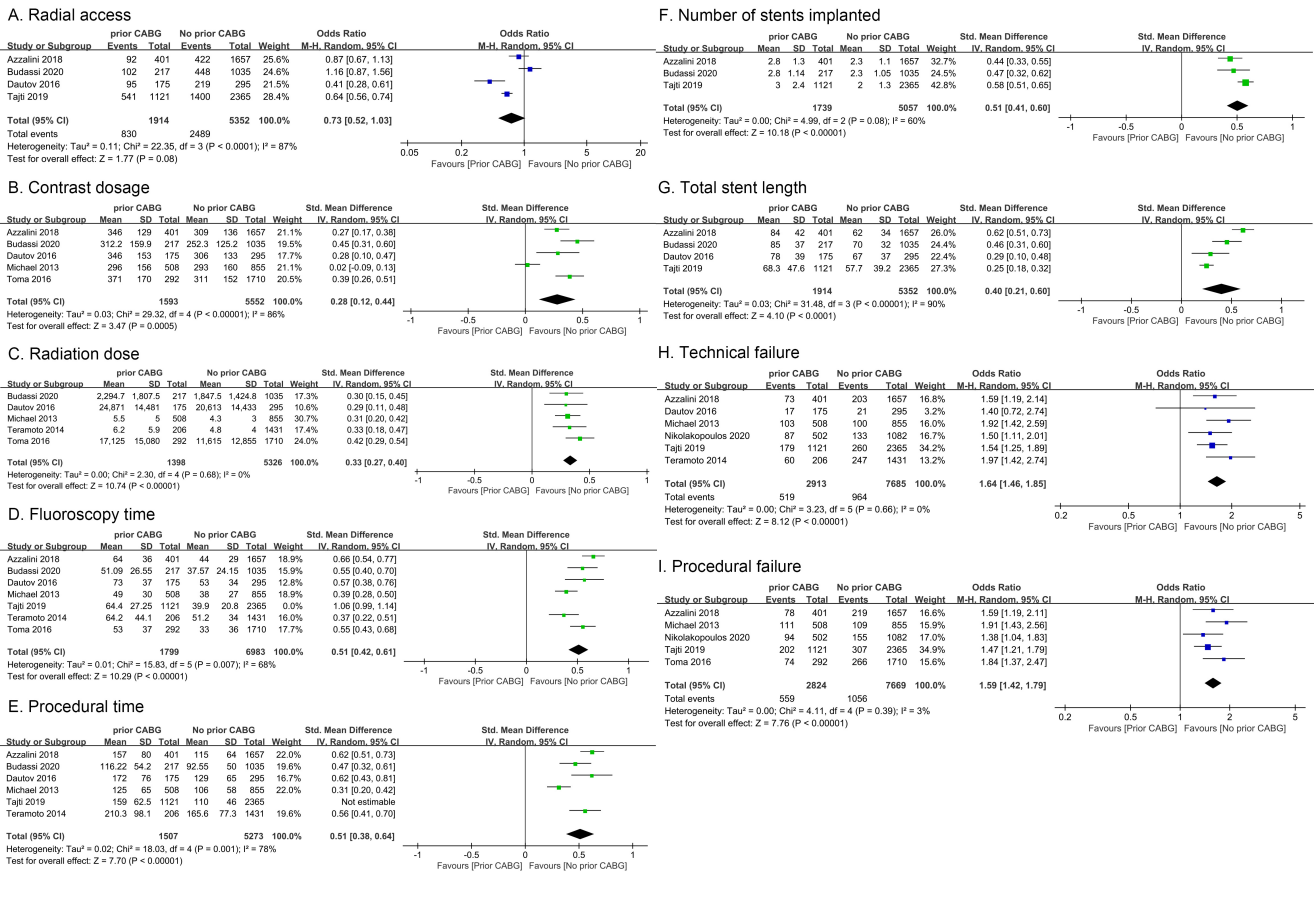
**Forest plots corresponding to procedural characteristics**. (A) Radial access. (B) Contrast dosage. (C) Radiation dose. (D) Fluoroscopy time. (E) Procedural time. (F) Number of stents implanted. (G) Total stent length. (H) Technical Failure. (I) Procedural failure. Risk ratios and pooled odds ratios with 95% CIs are displayed.

The proportion of radial access used in CTO-PCI was reported in four studies of 7266 patients, with high heterogeneity being detected for this endpoint (*$I^{2}$* = 87%, *p *< 0.001) [9, 10, 11, 13]. While a trend towards a lower radial access proportion was evident for individuals with a history of CABG, it was non-significant (OR = 0.73, 95% CI 0.52–1.03, *p* = 0.08; Fig. 2A). Heterogeneity changed little after analyzing by omitting one study in each turn.

#### 3.2.2 Contrast Dosage

Six studies including 10,631 patients reported on contrast dosages, with high heterogeneity for this endpoint (*$I^{2}$* = 86%, *p *< 0.001) [5, 9, 10, 11, 13, 14]. Overall, patients with a history of CABG consumed more contrast them patients without such a history (SMD = 0.28, 95% CI 0.12–0.44, *p *< 0.001; Fig. 2B). Tajti 2019 was excluded because the data of radiation dose in this study is not normally distributed. The heterogeneity of the results is mainly due to Micheal 2013 because this study is relatively old. The development of CTO-PCI technology in recent years has affected the operation time and the amount of contrast. *$I^{2}$* is decreased from 86% to 34% after this study is excluded and the conclusion did not change after adjustment.

#### 3.2.3 Radiation Dose and Fluoroscopy Time

Seven studies [5, 7, 9, 10, 11, 12, 14] including 12,268 patients assessed radiation dose and fluoroscopy time for the CTO-PCI procedure, revealing that individuals with a history of prior CABG exhibited both an increase in radiation dose (SMD = 0.33, 95% CI 0.27–0.40, *p *< 0.001, *$I^{2}$* = 0%, *p* = 0.68; Fig. 2C) and fluoroscopy time (SMD = 0.51, 95% CI 0.42–0.61, *p *< 0.001, *$I^{2}$* = 68%, *p *< 0.001; Fig. 2D). Azzalini 2018 and Tajti 2019 was excluded because the data of radiation dose in this study is not normally distributed [9, 13]. The heterogeneity in fluoroscopy time is mainly due to Micheal 2013 and Teramoto 2014 because these studies are relatively old [5, 7]. *$I^{2}$* is decreased from 68% to 0% after these studies are excluded and the conclusion did not change after adjustment.

#### 3.2.4 Procedural Duration

Six studies [5, 7, 9, 10, 11, 13] of 10,266 patients reported on procedural duration, with high heterogeneity being evident for this endpoint (*$I^{2}$* = 78%, *p *< 0.001). The overall procedural duration was longer for patients with a history of CABG (SMD = 0.51, 95% CI 0.38–0.64, *p *< 0.001; Fig. 2E). Tajti 2019 was excluded because the data of radiation dose in this study is not normally distributed. The heterogeneity of the results is mainly due to Micheal 2013 because this study is relatively old. *$I^{2}$* is decreased from 78% to 2% after this study is excluded and the conclusion did not change after adjustment.

#### 3.2.5 Number and Length of Implanted Stents

Implanted stent numbers and length were respectively reported by three [9, 10, 13] and four [9, 10, 11, 13] studies. Patients exhibiting a history of CABG were implanted with more stents on average (SMD = 0.51, 95% CI 0.41–0.60, *p *< 0.001, *$I^{2}$* = 60%, *p* = 0.08; Fig. 2F) and exhibited a longer average stent length (SMD = 0.40, 95% CI 0.21–0.60, *p *< 0.001, *$I^{2}$* = 90%, *p *< 0.001; Fig. 2G) relative to patients with no history of CABG. Heterogeneity changed little after analyzing by omitting one study in each turn.

#### 3.2.6 Technical and Procedural Failure Rates

Six studies [5, 7, 9, 11, 12, 13] enrolling 10,598 and 11,745 patients respectively reported on technical failure and procedural failure rates. Patients exhibiting a history of CABG exhibited higher rates of both technical failure (OR = 1.64, 95% CI 1.46–1.85, *p *< 0.001, *$I^{2}$* = 0%, *p* = 0.66; Fig. 2H) and procedural failure (OR = 1.59, 95% CI 1.42–1.79, *p *< 0.001, *$I^{2}$* = 3%, *p* = 0.39; Fig. 2I), relative to patients without such a history. Budassi 2021 [10] was excluded because procedural failure mentioned in that study did not meet the definition in our study. Little heterogeneity was found in this outcome.

### 3.3 Clinical Outcomes

#### 3.3.1 In-Hospital Mortality

**Fig. 3.**
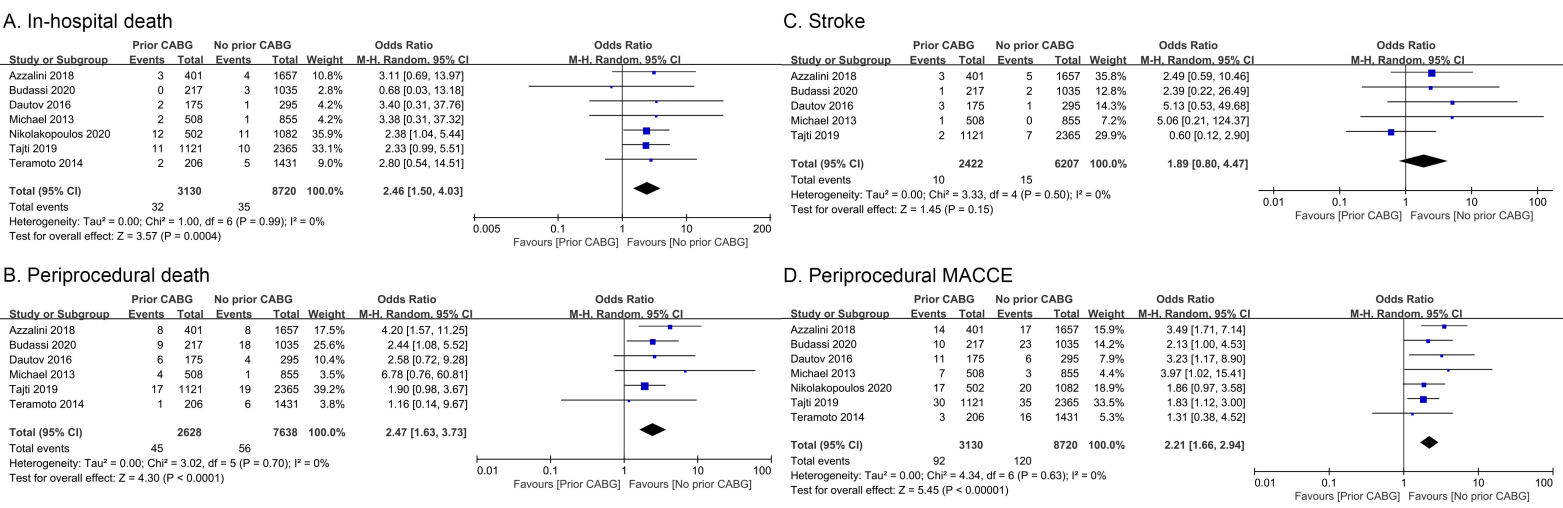
**Forest plots corresponding to clinical outcomes**. (A) In-hospital death. (B) Periprocedural death. (C) Stroke. (D) Periprocedural MACCE. Risk ratios and pooled odds ratios with 95% CIs are displayed.

Seven studies reported 67 instances of in-hospital mortality among 11,850 patients, with no heterogeneity among studies (*$I^{2}$* = 0%, *p *= 0.99) [5, 7, 9, 10, 11, 12, 13]. In total, the 3130 patients with a history of CABG exhibited 32 instances of in-hospital mortality, while the 8720 patients without such a history exhibited 35 such events. Accordingly, the pooled risk of in-hospital mortality was higher for individuals with a history of CABG (OR = 2.46, 95% CI 1.50–4.03, *p *< 0.001; Fig. 3A).

#### 3.3.2 Periprocedural Mortality

Six studies reported 101 instances of periprocedural mortality among 10,266 patients, with no evidence of heterogeneity (*$I^{2}$* = 0%, *p *= 0.70) [5, 7, 9, 10, 11, 13]. Of these events, 45 occurred among 2628 CABG patients while 56 occurred among 7638 patients without such a history. As such, periprocedural death rates were significantly elevated in patients with a history of prior CABG (OR = 2.47, 95% CI 1.63–3.73, *p *< 0.001; Fig. 3B).

#### 3.3.3 Stroke Incidence

In total, 25 instances of stroke were reported among 8629 patients in five studies [5, 9, 10, 11, 13], with no significant heterogeneity (*$I^{2}$* = 0%, *p *= 0.50). Of these events, 10 and 15 respectively occurred in patients with and without a history of CABG (n = 2422 and n = 6207, respectively). No significant differences are stroke incidence was evident when comparing these groups (OR = 1.89, 95% CI 0.80–4.47, *p* = 0.15; Fig. 3C).

#### 3.3.4 Periprocedural MACCE Incidence

Overall, seven studies [5, 7, 9, 10, 11, 12, 13] assessed MACCE incidence as a composite endpoint, including the incidence of procedure-related stroke, myocardial infarction, urgent target vessel revascularization, tamponade necessitating either pericardiocentesis or surgery, and death. This endpoint was not subject to any significant heterogeneity (*$I^{2}$* = 0%, *p *= 0.63). In total, 92 events were reported in 3130 CABG patients, while 120 were reported in 8720 patients without a history of CABG. Periprocedural MACCE incidence was significantly increased in patients with a history of CABG relative to patients that had not undergone CABG (OR = 2.21, 95% CI 1.66–2.94, *p *< 0.001; Fig. 3D).

### 3.4 Publication Bias Analyses

**Fig. 4.**
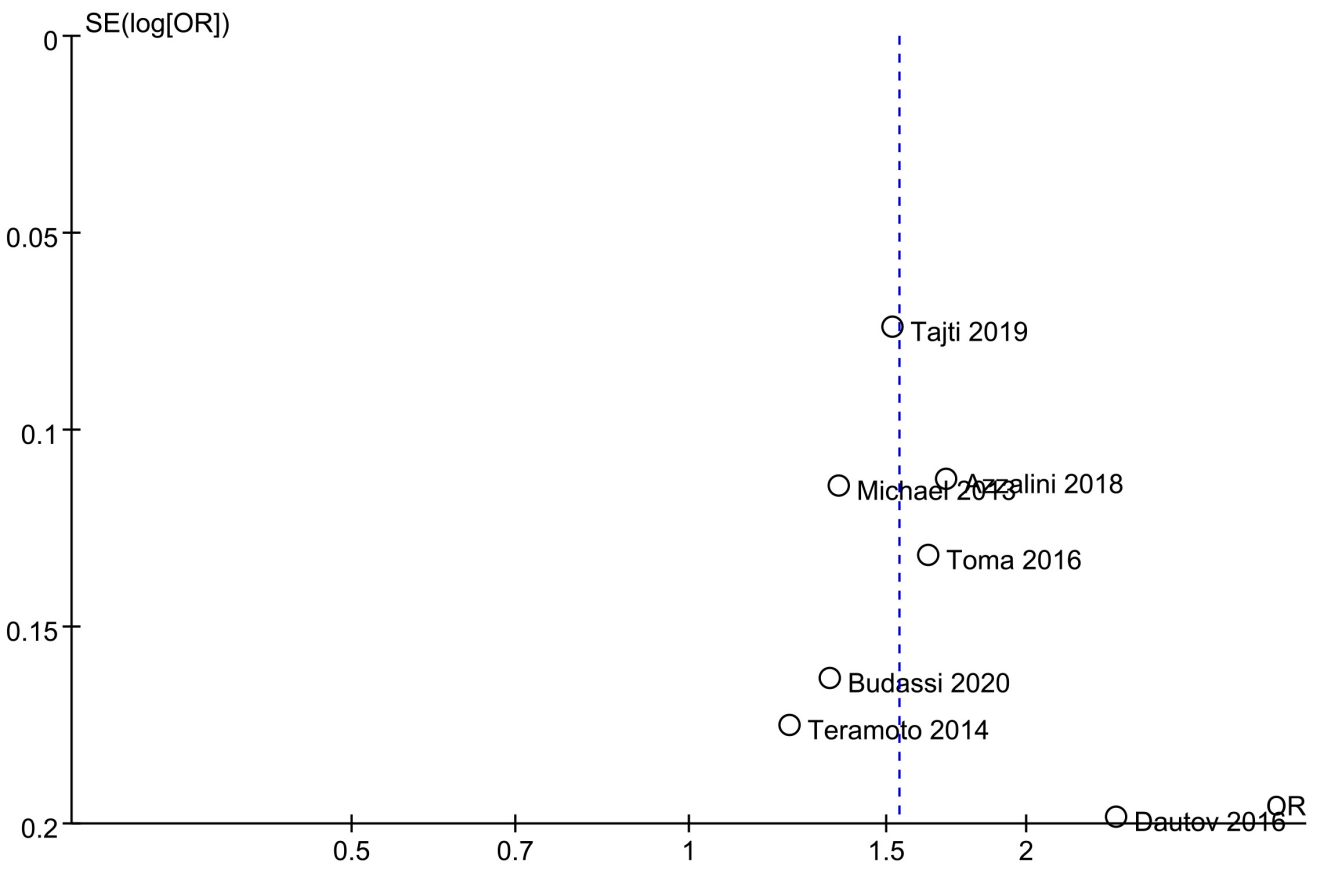
**Funnel plot corresponding to publication bias**.

Funnel plots were used to assess potential publication bias, and exhibited slight asymmetry consistent with the potential for reporting bias, as smaller studies exhibited larger treatment effects (Fig. 4). The reliability of the evidence for each study outcome was assessed, as summarized in **Supplementary Tables 1,2**.

## 4. Discussion

This is the largest meta-analysis conducted to date comparing CTO-PCI procedural characteristics and clinical outcomes for patients with a history of CABG, assessing pooled data from 13,509 patients in 8 observational studies. The accumulation of further data has afforded greater statistical power such that these risk estimates are more precise and reliable than those in prior studies [5, 7, 8, 9, 10, 11, 12, 13, 14].

Here, procedural characteristics and clinical outcomes associated with the CTO-PCI procedure were compared as a function of whether or not patients had previously undergone CABG, ultimately revealing that patients with such a history exhibited increases in contrast dosage, radiation dose, fluoroscopy time, and both the number and total length of stents used for the CTO-PCI procedure as compared to patients without such a history, whereas radial access was less frequently utilized for these patients. Moreover, a history of CABG was associated with elevated rates of MACCE, stroke, periprocedural mortality, and in-hospital mortality.

These findings suggest that the CTO-PCI procedure may be more challenging when performed in individuals that have previously undergone CABG, contributing to lower rates of success and poorer clinical outcomes as compared to those in patients without such a medical history. The higher radiation dose, operative duration, contrast dosage, and rates of procedural failure attest to the increased difficulty of PCI in CABG patients while also suggesting that the CTO lesions in these patients may exhibit greater complexity. This may in part be attributable to the fact that CABG is the favored revascularization approach employed in patients exhibiting complex anatomical characteristics. Moreover, a history of prior CABG is associated with negative vascular remodeling and more rapid atherosclerotic disease progression following this procedure, resulting in further increases in procedural complexity. In a prior meta-analysis, we found CABG patients undergoing CTO-PCI to exhibit more complex lesion characteristics including higher J-CTO scores, longer lesion length, greater levels of calcification, and more proximal cap ambiguity [15]. These results are shown in **Supplementary Fig. 1**. The Poorer clinical outcomes in individuals with a history of CABG may be attributable to their being older, exhibiting more comorbidities, and more frequently experiencing procedure-related complications. Our prior meta-analysis found prior CABG to be related to higher rates of procedural complications such as contrast-induced nephropathy, major bleeding, and perforation. A large cohort study of 2058 patients from Canada, Europe, and the USA published by Azzalini *et al*. [9] found lower rates of procedural success rates and higher rates of in-hospital complications in individuals with a history of CABG, with increased rates of target vessel failure of a medial 1-year follow-up period.

This study is subject to certain limitations. For one, all included studies were observational studies and they are thus susceptible to unknown confounds. Secondly, little long-term follow-up data were available for patients in either group. Thirdly, the heterogeneity of at least some of the studies included and the different time periods of the study which may influence the results [5, 7, 9, 10, 11, 12, 13, 14]. Lastly, the CTO-PCI technique has improved rapidly in recent years owing to technological and operative advances, and as such study age may have a significant impact on the associated procedural characteristics and clinical outcomes.

## 5. Conclusions

In summary, this meta-analysis suggests that CTO-PCI procedures may be more challenging and associated with worse outcomes when performed in patients that have undergone prior CABG, with these patients also exhibiting poorer in-hospital MACCE incidence and mortality rates relative to patients without a history of CABG.

## Supplementary Material

Supplementary material associated with this article can be found, in the online version, at https://doi.org/10.31083/j.rcm2403089.
