# Supplementary material for: Procedural Characteristics and Clinical Outcomes Associated with Chronic Total Occlusion Percutaneous Coronary Intervention in Patients with a History of Prior Coronary Artery Bypass Graft: A Meta-Analysis
Source: Rev Cardiovasc Med. 2023 Mar 13;24(3):89. doi: 10.31083/j.rcm2403089 (PMC11263990; doi:10.31083/j.rcm2403089)

Table 1

| **Procedural characteristics** | | | | | |
| --- | --- | --- | --- | --- | --- |
| **Outcomes** | **No of Participants (studies)** | **Quality of the evidence** (GRADE) | **Relative effect (95% CI)** | **Anticipated absolute effects** | |
|  |  |  |  |  | |
|  |  |  |  | **Risk with Control** | **Risk difference with Prior CABG** (95% CI) |
| **Radial access** | 7266 (4 studies) | ⊕⊕⊝⊝ **LOW** | **OR 0.73**  (0.52 to 1.03) | **Study population** | |
|  |  |  |  | **465 per 1000** | **77 fewer per 1000** (from 154 fewer to 7 more) |
|  |  |  |  | **Moderate** | |
|  |  |  |  | **512 per 1000** | **78 fewer per 1000** (from 159 fewer to 7 more) |
| **Contrast volume** | 10631 (6 studies) | ⊕⊕⊝⊝ **LOW** |  |  | The mean contrast volume in the intervention groups was **0.25 standard deviations higher** (0.13 to 0.37 higher) |
| **Radiation dose** | 12268 (7 studies) | ⊕⊕⊝⊝ **LOW** |  |  | The mean radiation dose in the intervention groups was **0.3 standard deviations higher** (0.21 to 0.4 higher) |
| **Fluoroscopy time** | 12268 (7 studies) | ⊕⊝⊝⊝ **VERY LOW**^1^ due to imprecision |  |  | The mean fluoroscopy time in the intervention groups was **0.59 standard deviations higher** (0.37 to 0.82 higher) |
| **Procedure time** | 10266 (6 studies) | ⊕⊕⊝⊝ **LOW** |  |  | The mean procedure time in the intervention groups was **0.59 standard deviations higher** (0.37 to 0.81 higher) |
| **No. of stents implanted** | 6796 (3 studies) | ⊕⊕⊝⊝ **LOW** |  |  | The mean no. of stents implanted in the intervention groups was **0.51 standard deviations higher** (0.41 to 0.6 higher) |
| **Total stent length** | 7266 (4 studies) | ⊕⊝⊝⊝ **VERY LOW**^1^ due to imprecision |  |  | The mean total stent length in the intervention groups was **0.4 standard deviations higher** (0.21 to 0.6 higher) |
| **Technical failure** | 10598 (6 studies) | ⊕⊕⊝⊝ **LOW** | **OR 1.64**  (1.46 to 1.85) | **Study population** | |
|  |  |  |  | **125 per 1000** | **65 more per 1000** (from 48 more to 84 more) |
|  |  |  |  | **Moderate** | |
|  |  |  |  | **120 per 1000** | **63 more per 1000** (from 46 more to 81 more) |
| **Procedural failure** | 11745 (6 studies) | ⊕⊕⊝⊝ **LOW** | **OR 1.77**  (1.44 to 2.17) | **Study population** | |
|  |  |  |  | **135 per 1000** | **81 more per 1000** (from 48 more to 118 more) |
|  |  |  |  | **Moderate** | |
|  |  |  |  | **131 per 1000** | **80 more per 1000** (from 47 more to 115 more) |
| *The basis for the **assumed risk** (e.g. the median control group risk across studies) is provided in footnotes. The **corresponding risk** (and its 95% confidence interval) is based on the assumed risk in the comparison group and the **relative effect** of the intervention (and its 95% CI).  **CI:** Confidence interval; **OR:** Odds ratio; | | | | | |
| GRADE Working Group grades of evidence **High quality:** Further research is very unlikely to change our confidence in the estimate of effect.  **Moderate quality:** Further research is likely to have an important impact on our confidence in the estimate of effect and may change the estimate. **Low quality:** Further research is very likely to have an important impact on our confidence in the estimate of effect and is likely to change the estimate. **Very low quality:** We are very uncertain about the estimate. | | | | | |
| ^1^ Different definition | | | | | |

Table 2

| **Clinical outcomes** | | | | | |
| --- | --- | --- | --- | --- | --- |
| **Outcomes** | **No of Participants (studies)** | **Quality of the evidence** (GRADE) | **Relative effect (95% CI)** | **Anticipated absolute effects** | |
|  |  |  |  |  | |
|  |  |  |  | **Risk with Control** | **Risk difference with Prior CABG** (95% CI) |
| **In-hospital death** | 11850 (7 studies) | ⊕⊕⊝⊝ **LOW** | **OR 2.46**  (1.5 to 4.03) | **Study population** | |
|  |  |  |  | **4 per 1000** | **6 more per 1000** (from 2 more to 12 more) |
|  |  |  |  | **Moderate** | |
|  |  |  |  | **3 per 1000** | **4 more per 1000** (from 1 more to 9 more) |
| **Periprocedural myocardial infarction** | 10266 (6 studies) | ⊕⊕⊝⊝ **LOW** | **OR 2.47**  (1.63 to 3.73) | **Study population** | |
|  |  |  |  | **7 per 1000** | **11 more per 1000** (from 5 more to 19 more) |
|  |  |  |  | **Moderate** | |
|  |  |  |  | **6 per 1000** | **9 more per 1000** (from 4 more to 16 more) |
| **Stroke** | 8629 (5 studies) | ⊕⊕⊝⊝ **LOW** | **OR 1.89**  (0.8 to 4.47) | **Study population** | |
|  |  |  |  | **2 per 1000** | **2 more per 1000** (from 0 fewer to 8 more) |
|  |  |  |  | **Moderate** | |
|  |  |  |  | **3 per 1000** | **3 more per 1000** (from 1 fewer to 10 more) |
| **MACCE** | 11850 (7 studies) | ⊕⊕⊝⊝ **LOW** | **OR 2.21**  (1.66 to 2.94) | **Study population** | |
|  |  |  |  | **14 per 1000** | **16 more per 1000** (from 9 more to 26 more) |
|  |  |  |  | **Moderate** | |
|  |  |  |  | **15 per 1000** | **18 more per 1000** (from 10 more to 28 more) |
| *The basis for the **assumed risk** (e.g. the median control group risk across studies) is provided in footnotes. The **corresponding risk** (and its 95% confidence interval) is based on the assumed risk in the comparison group and the **relative effect** of the intervention (and its 95% CI).  **CI:** Confidence interval; **OR:** Odds ratio; | | | | | |
| GRADE Working Group grades of evidence **High quality:** Further research is very unlikely to change our confidence in the estimate of effect.  **Moderate quality:** Further research is likely to have an important impact on our confidence in the estimate of effect and may change the estimate. **Low quality:** Further research is very likely to have an important impact on our confidence in the estimate of effect and is likely to change the estimate. **Very low quality:** We are very uncertain about the estimate. | | | | | |
|  |  |  |  |  |  |

Fig 1. Forest plots corresponding to angiographic characteristics. A. Blunt stump; B. Moderate or severe calcification; C. Bending; D. Lesion length > 20mm; E. Previous failed attempt; F. J-CTO score. Risk ratios and pooled odds ratios with 95% CIs are displayed.

Figure 1 A


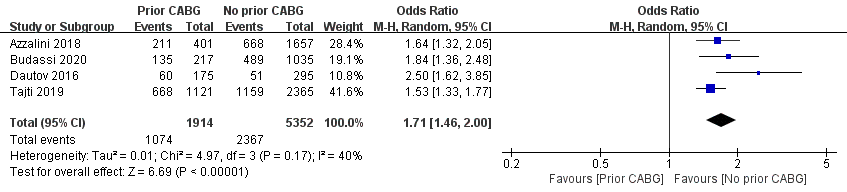


Figure 1 B


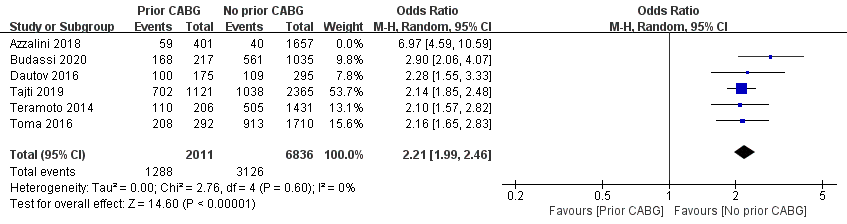


Figure 1 C


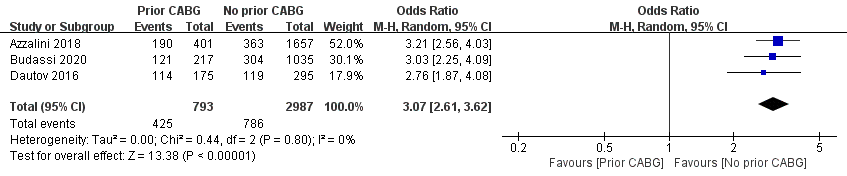


Figure 1 D


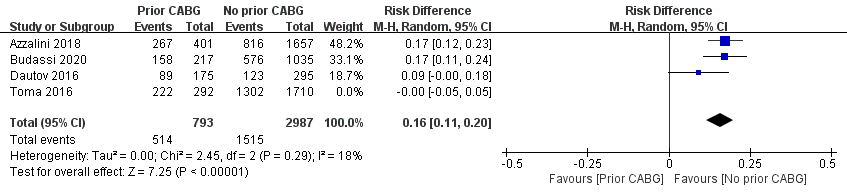


Figure 1 E


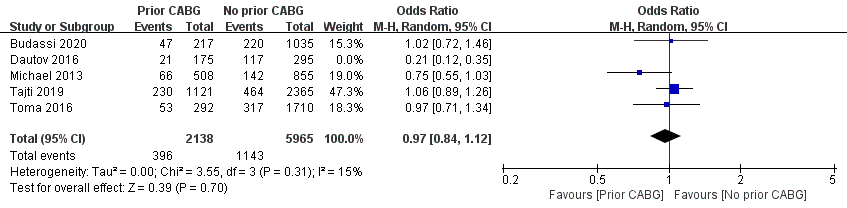


Figure 1 F


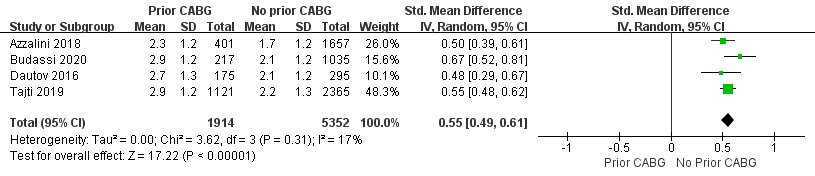

Supplement: Supplementary file 1 [file 2153-8174-24-3-089-s1.zip › 2153-8174-24-3-089-s1/Supplementary materials.docx]
